# Electrical spiking of psilocybin fungi

**DOI:** 10.1080/19420889.2022.2136118

**Published:** 2022-10-24

**Authors:** Antoni Gandia, Andrew Adamatzky

**Affiliations:** a Institute for Plant Molecular and Cell Biology, Valencia, ES, Spain; b Unconventional Computing Laboratory, UWE, Bristol, UK

**Keywords:** Psilocybin fungi, electrical activity, spiking

## Abstract

Psilocybin fungi, aka “magic” mushrooms, are well known for inducing colorful and visionary states of mind. Such psychoactive properties and the ease of cultivating their basidiocarps within low-tech setups make psilocybin fungi promising pharmacological tools for mental health applications. Understanding of the intrinsic electrical patterns occurring during the mycelial growth can be utilized for better monitoring the physiological states and needs of these species. In this study we aimed to shed light on this matter by characterizing the extra-cellular electrical potential of two popular species of psilocybin fungi: Psilocybe *tampanensis* and *P. cubensis*. As in previous experiments with other common edible mushrooms, the undisturbed fungi have shown to generate electric potential spikes and trains of spiking activity. This short analysis provides a proof of intrinsic electrical communication in psilocybin fungi, and further establishes these fungi as a valuable tool for studying fungal electro-physiology.

## 1. Introduction

Psilocybin fungi, popularly known as “magic” mushrooms, are a group of different species of psychoactive basidiomycetes that have gained an immense popularity since the ethnomycologists Gordon Wasson and his wife Valentina Pavlovna Wasson introduced them to the Western audiences in 1957 [1–3]. Psilocybin fungi are remarkably famous for inducing mystical-type experiences thanks to tryptamine alkaloids contained in its hyphae, mainly psilocybin, baeocystin and norbaeocystin [4,5], to which the community of users and a growing pool of scientific evidence grant different potential benefits, such as treating depression to helpmanage alcoholism and drug addiction [6–11].

These organisms have been ever since surrounded by an aura of mysticism and criticism in equal shares, with opinions mostly tied to religious or political beliefs rather than being based in scientific research. Nevertheless, magic mushrooms have been used by different human cultures across the globe for millennia, probably since the dawn of mankind, as a tool for exploring and healing psychological and physical disorders, or simply to inspire awe, creativity, introspection, and a better appreciation for nature [12–18]. Considering their cultural and psycho-pharmaceutical importance, the scientific community is trying to make sense of different aspects of their ecology, physiology, pharmacology, and overall, potential biotechnological applications favoring human society and Earth’s biosphere.

Recent research suggests that spontaneous electrical low-frequency oscillations (SELFOs) are found across most organisms on Earth, from bacteria to humans, including fungi, playing an important role as electrical organization signals that guide the development of an organism [19]. Considering its potential function as communication and integration waves, detecting and translating SELFOs in psilocybin fungi species may prove to be a great contribution in understanding the growth and behavior of these organisms, a knowledge that could be added to the toolbox of cultivation and pharmacological optimization techniques used by the fungal biotech industry.

Thereby, we recorded the extracellular electrical potential in mushrooms and mycelium-colonized substrates as indicators of the fungi intrinsic activity. Action potential-like spikes of electrical potential have been observed using intra-cellular recording of mycelium of *Neurospora crassa* [20] and further confirmed in intra-cellular recordings of action potential in hyphae of *Pleurotus ostreatus* and *Armillaria gallica* [21] and in extra-cellular recordings of basidiocarps of and substrates colonized by mycelium of *P. ostreatus* [22], *Ganoderma resinaceum* [23], and *Omphalotus nidiformis, Flammulina velutipes, Schizophyllum commune*

**Figure 1.**
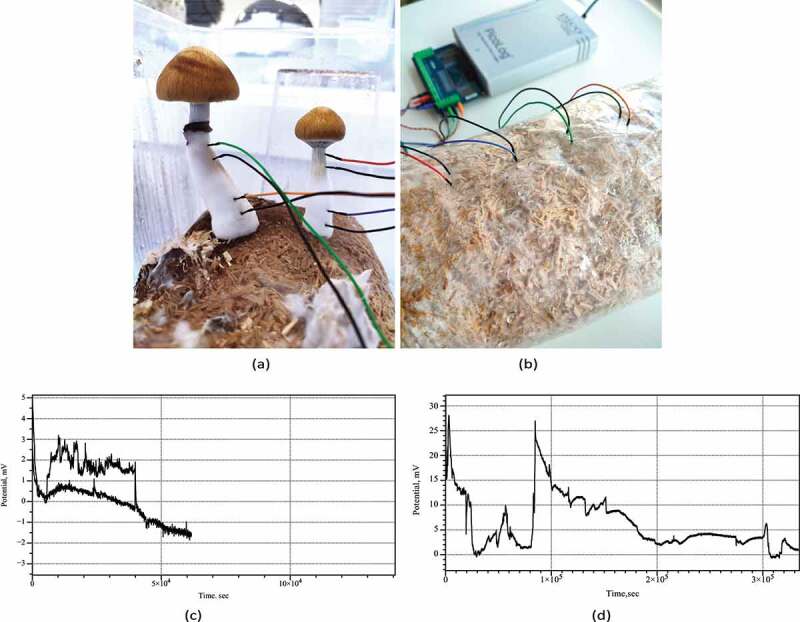
Experimental setup. (a) Example of recording from *Psilocybe cubensis* basidiocarps. (b) Example of recording from *Psilocybe tampanensis* mycelium-colonized substrate and view of the experimental setup, in which the electrodes with cables and Pico ADC-24 are seen. (cd) Examples of electrical activity of (c) *Psilocybe cubensis*, two channels, and (d) *Psilocybe tampanensis*, one channel.

and *Cordyceps militaris* [24]. While the exact nature of the traveling spikes remains uncertain we can speculate, by drawing analogies with oscillations of electrical potential of slime mold *Physarum poly- cephalum* [25–28], that the spikes in fungi are triggered by calcium waves, reversing of cytoplasmic flow, and translocation of nutrients and metabolites.

The paper is structured as follows. First, we present the experimental setup in Sect. 2. Secondly, in Sect. 3 we analyzed the electrical activity of the fungi. Finally, section 4 presents some conclusions and directions for further research.

## 2. Methods

Two widely distributed species of psilocybin fungi, namely *Psilocybe cubensis* strain “B+” (Mondo Myco- logicals BV, NL), and *Psilocybe tampanensis* strain “ATL7” (Mimosa Therapeutics BV, NL), were cultured separately on a mixture of hemp shavings amended with 5% wheat flour, at 60% moisture content, in polypropylene (PP5) filter-patch bags.^1^ Electrical activity of the basidiocarps and the colonized substrate was recorded using pairs of iridium-coated stainless steel sub-dermal needle electrodes (Spes Medica S.r.l.,).

^1^Experiments were conducted at Mimosa Therapeutics BV, The Netherlands.

Italy), with twisted cables and ADC-24 (Pico Technology, UK) high-resolution data logger with a 24-bit analog-to-digital converter (Figure 1). Resistance of electrodes with cables was 1 Ohm. Input impedance of Pico ADC-24 logger was 2 MOhm, ADC input bias current less than 50 nA.

We recorded electrical activity at one sample per second. During the recording, the logger has been doing as many measurements as possible (typically up to 600 per second) and saving the average value. We set the acquisition voltage range to 156 mV with an offset accuracy of 9 *µ*V at 1 Hz to maintain a gain error of 0.1%. Each electrode pair was considered independent with the noise-free resolution of 17 bits and conversion time of 60 ms. Each pair of electrodes, called channels, reported a difference of the electrical potential between the electrodes. Distance between electrodes was 1–2 cm. We have conducted eight experiments, in each experiments we recorded electrical activity of the fungi via four channels, i.e. 32 recordings in total.

## 3. Results

Electrical activity recorded from both species of psilocybin fungi shows a rich dynamics of electrical potential. Examples of the recording conducted for nearly four days are shown in Figure 1c-d. Drift of the base potential can be up to 10–15 mV however rate of the base potential change is measured in days therefore it does affect our ability to recognize spikes. Plots also show that in some cases the signal-to-noise ratio might be substantially low. We omitted such cases from the spike detection pool.

**Figure 2.**
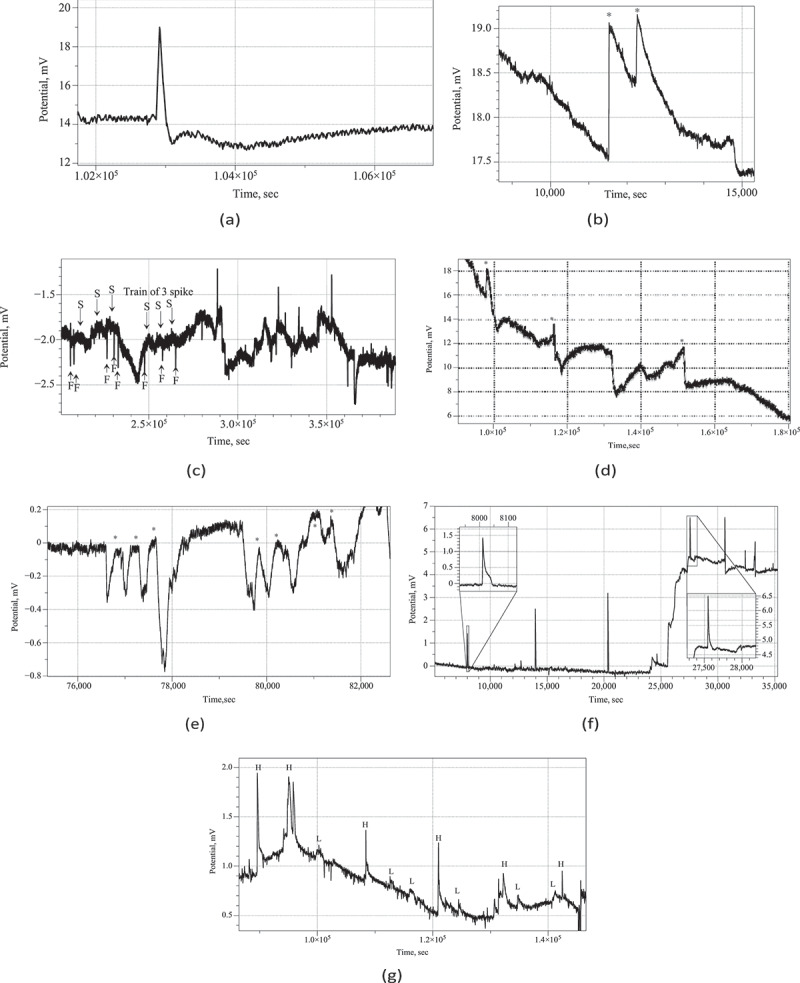
(a) Example of an action-potential like voltage spike, *Psilocybe cubensis*. (b) Train of two spikes, *Psilocybe cubensis*(c) Example of fast and slow spike activity,*Psilocybe cubensis*. An average duration of a fast spike is 3 min. An average duration of a slow spike is 16 min. Examples of fast spikes are labeled ‘F’ and slow ‘S’. Train of three slow spikes is also marked. (d) Example of three spikes in electrical potential of *Psilocybe tampanensis*, peaks of the spike are labeled ⋆. (e) Twotrains of spikes recorded in *Psilocybe cubensis*: one train comprises of three spikes, another of four spikes; spike are marked by ⋆.(f) Very fast, average 1.5 min, spikes of electrical potential recorded in *Psilocybe cubensis*. (g) Co-existence of high amplitude,labeled ‘H’, and low amplitude, labeled ‘L’, spikes in electrical activity of *Psilocybe cubensis.*

We observed action-potential like spikes of electrical potential. Most expressive spikes, see e.g. Figure 2a, show very characteristics of action potential recorded in nervous system with distinctive depolarization and repolarization phases and a refractory period. In the exemplar action-potential like spike shown in Figure 2a depolarization phase is c. 18 sec up to 4.5 mV; re-polarization phase is 97 sec; refractory period is rather long c. 450 sec.

In some cases, as illustrated in Figure 2b, two action-potential like spikes can occur at so short interval that they almost merge. In this particular example, an average spike duration is 13 min, and average amplitude is 1.4 mV.

More commonly the spikes emerge in the trains of spikes. A train is a sequence of spikes where distance between two consecutive spikes does not exceed an average duration of a spike. Two trains of spikes are shown in Figure 2e. Also, spike can stand alone, as shown in Figure 2d.

Amongst many types of spikes classed by their duration we can select very fast spikes, with duration of 1–2 min, and slow spikes, whose width can be 15–60 min. An example of very fast spikes directly co-existing with slow spikes is shown in Figure 2c. In some cases, only very fast spikes can be observed during the whole duration of the recording, see an example in Figure 2f.

A co-existence of spikes with high, 0.5–1 mV, and low, 0.1–0.3 mV, is evidenced in the recording plotted in Figure 2g. An amplitude, however, might be not a good characteristic of spikes because it only indicated how far away a wave-front of propagating electrical activity was from a pair of differential electrodes.

**Figure 3.**
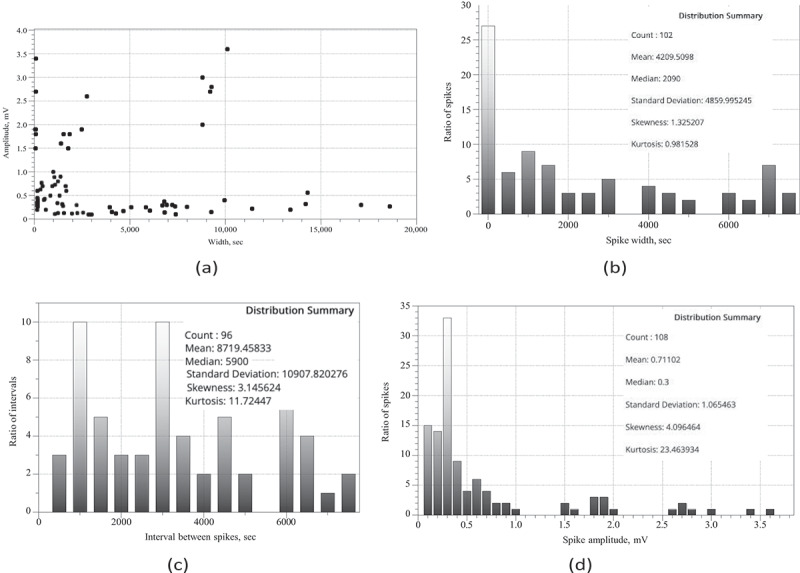
(a) Spike width versus spike amplitude distribution constructed on recording from bother species of fungi studied. (b) Distribution of spike widths, (c) Distribution of interval between spikes. Bin size is 500 in both distributions. (d) Distribution of spike amplitudes, bin size is 0.1.

Distribution of spike amplitudes version spike width is shown in Figure 3a. Pearson correlation *R* = 0.0753 calculated on the distribution is technically a positive correlation, however its low value shows that the relationship between spike width and amplitude is weak.

Distributions of spike width (Figure 3b), intervals between spikes (Figure 3c) and spike amplitudes (Figure 3d) are not normal. This is demonstrated by Kolmogorov-Smirnov test of normality. Values of Kolmogorov- Smirnov statistic are 0.19467 for width distribution, 0.21914 for interval distribution, and 0.28243 for amplitude distribution. Corresponding p-values are 0.00072, 0.00016 and 0.00001.

Integrative parameters of spiking behavior are shown in Table 1. Average duration of a spike is 70 min (*σ *= 81 min), median duration is 35 min. Average amplitude of a spike is 0.71 mV (*σ *= 1.06 mV), median is 0.3 mV. Average distance between spikes is 145 min (*σ *= 181 min), median is 98 min. That is average/median distance between two spikes is a double of the average/median duration of a spike. By the definition of the train, this means that most spikes observed are solitary spikes. Standard deviations of spike duration and amplitude and of interval between spikes are higher than respective average values. This indicates data are more spread out and we should look out for distinct families of spikes.

**Table 1.** Statistical parameters of spiking. In each cell we show average, standard deviation and median.

Species	Duration, sec	Amplitude, mV	Distance, sec
Over all species	4209, 4859, 2090	0.71, 1.06, 0.3	8719, 10,907, 5900
*Psilocybe tampanensis*	6246, 4196, 8800	2.33, 2.22, 1.71	17,566, 11,183, 17,300
*Psilocybe cubensis*	4082, 4892, 1640	0.48, 0.56, 0.30	8000, 10,458, 4930
*P. cubensis*, fast spikes (1–3 min)	148, 38, 165	0.72, 0.83, 0.35	6128, 4516, 3955
*P. cubensis*, slow spikes (up to 6 hr du-	1408, 274, 1394	0.68, 0.60, 0.50	3243, 2125, 3105
ration)			
*P. cubensis*, very slow spikes (up to 2	7943, 4658, 6810	0.24, 0.11, 0.25	11,948, 13,639, 7880
days)			

Let us separate species and – if any – families of spikes in each species. In *Psilocybe tampanensis* spikes are relatively uniform (Table 1): average duration 104 min with *σ *= 69 min, average distance between spikes is over three hours and median distance equal to average. In *Psilocybe cubensis* we propose three families of spikes: fast spike, up to 3 min duration, slow spikes, up to 6 hr duration, and very slow spikes, up to 2

days (Table 1). Fast spikes rarely form train, an average distance between fast spikes is 17 hr. An average duration of a slow spike is 3.9 hr with an average distance between slow spikes is 9 hr. Average amplitudes of fast and slow spikes are comparable, 0.72 mV and 0.68 mV, respectively. An average duration of a very slow spike of *Psilocybe cubensis* is 22 hr with an average distance between spikes of 33 hr. The very slow spikes have, comparatively to fast and slow spikes, low amplitude of 0.24 mV in average.

## 4. Discussion

We found that psilocybin fungi exhibit a rich spectrum of oscillations of extracellular electrical potential. We illustrated several types of oscillations and characterized families of fast, slow and very slow oscillations. We demonstrated that several scales – minutes, hours and day – of oscillators states co-exist in basidiocarps and mycelium network of psilocybin fungi. This co-existence is similar to electrical oscillation of a human brain, where fast oscillations might be related to responses to stimulation, including endogenous stimulation by release of nutrients, and slow oscillations might be responsible for memory consolidation [29]. Future research could be concerned with decoding and understanding the spiking events to monitor growth, development and physiological states and overall condition of the fungi both in cultivation setups and natural environments. If we were able to decode spiking patterns of fungi we would be able to ’speak back’ to the mycelial network to manipulate the network’s morphology, behavior and, potentially, enhance production of basidiocarps and sclerotia.
